# Management of epigenomic networks entailed in coronavirus infections and COVID-19

**DOI:** 10.1186/s13148-020-00912-7

**Published:** 2020-08-05

**Authors:** Ranim El Baba, Georges Herbein

**Affiliations:** 1 Department Pathogens & Inflammation-EPILAB, UPRES EA4266, University of Franche-Comté and University of Bourgogne Franche-Comté, F-25030 Besançon, France; 2 Université Libanaise, Beirut, Lebanon; 3 Department of Virology, CHRU Besancon, F-25030 Besançon, France

**Keywords:** Coronavirus, SARS-CoV, MERS-CoV, SARS-CoV-2, COVID-19, Epigenetic, Inflammation

## Abstract

Coronaviruses (CoVs) are highly diverse single-stranded RNA viruses owing to their susceptibility to numerous genomic mutations and recombination. Such viruses involve human and animal pathogens including the etiologic agents of acute respiratory tract illnesses: severe acute respiratory syndrome coronavirus (SARS-CoV), Middle East respiratory syndrome coronavirus (MERS-CoV), and the highly morbific SARS-CoV-2. Coronavirus disease 2019 (COVID-19), an emerging disease with a quick rise in infected cases and deaths, was recently identified causing a worldwide pandemic. COVID-19 disease outcomes were found to increase in elderly and patients with a compromised immune system. Evidences indicated that the main culprit behind COVID-19 deaths is the cytokine storm, which is illustrated by an uncontrolled over-production of soluble markers of inflammation. The regulation process of coronavirus pathogenesis through molecular mechanism comprise virus-host interactions linked to viral entry, replication and transcription, escape, and immune system control. Recognizing coronavirus infections and COVID-19 through epigenetics lens will lead to potential alteration in gene expression thus limiting coronavirus infections. Focusing on epigenetic therapies reaching clinical trials, clinically approved epigenetic-targeted agents, and combination therapy of antivirals and epigenetic drugs is currently considered an effective and valuable approach for viral replication and inflammatory overdrive control.

## Background

Coronaviruses are non-segmented, enveloped viruses with a positive-sense single-stranded RNA genome belonging to Coronaviridae family [1–3]. CoVs share similar genome organization, but differ phenotypically and genotypically [4, 5]. High frequency of RNA recombination, RNA-dependent RNA polymerase (RdRp) fickleness, and the bulky genomes for RNA viruses are considered leading factors for CoVs’ diversity [5]. Humans are infected by seven CoVs, including HCoV-229E and HCoV-NL63 belonging to Alphacoronavirus; HCoV-OC43 and HCoV HKU1 belonging to Betacoronavirus lineage A; these four viruses are known to be endemic [4–6]. Three human coronaviruses (HCoVs) caused epidemics expressing high morbidity and mortality rates: SARS-CoV belonging to Betacoronavirus lineage B, MERS-CoV or HCoV-EMC belonging to Betacoronavirus lineage C, and the 2019 novel coronavirus 2019-nCoV/SARS-CoV-2 [6–8].

SARS-CoV emerged in Guangdong Province, China, in February, 2003 [9, 10]. It resulted in 8098 human infections and 774 deaths, and it disseminated into 37 countries [3, 11]. In 2012, MERS-CoV was initially detected in the Kingdom of Saudi Arabia revealing 2494 confirmed infected cases and 858 mortalities. It was spread to 27 additional countries [3, 12]. While the MERS-CoV outbreak has been mostly limited to the Middle Eastern region, it is likely that more re-emerging HCoVs might endanger the global communal health condition. SARS-CoV-2 was identified in late December, 2019 in Wuhan, China [8]. The World Health Organization (WHO) declared that COVID-19 was listed as the sixth Public Health Emergency of International Concern (PHEIC), implicating that it may pose risks to various countries and entail an international response [8, 13, 14]. A situation report showed COVID-19 data as received by WHO in 9 June 2020: 7,039,918 confirmed cases and 404,396 deaths were globally reported in American, European, Eastern Mediterranean, Western Pacific, South-East Asia, and African regions [15]. However, underestimating COVID-19’s burden was due to the fact that patients with mild COVID-19 symptoms or asymptomatic patients might not seek medical care for proper diagnosis.

As outbreaks can ensue rapidly worldwide, it is quite necessary to emphasize on novel therapeutic approaches. Although investment in biomedical and pharmaceutical research has increased significantly, the annual number of new treatments approved by the Food and Drug Administration (FDA) has remained relatively limited [11, 16]. Generally, the available treatment strategies for emerging coronavirus’ strains, that led to significant pandemics, are inadequate to effectively advance patient’s outcome [17]. These strategies have been less successful for RNA viruses compared to DNA viruses as the former mutates at a higher rate resulting in drug resistance [4]. Yet, HCoVs potentially influence the host’s epigenome, and this will aid in discovering new targets for therapeutic interventions to gain more insights for the development of antiviral therapeutics and vaccines [9, 18]. The primary objective of this review is to evaluate the epigenetic mechanisms involved in HCoVs’ infection and to highlight on epigenetic therapies in order to reduce peak incidence and global deaths resulting from HCoVs’ outbreaks worldwide.

### Epigenetic mechanisms at work in coronavirus replication

#### Epigenetic regulation of coronavirus replication

**Fig. 1:**
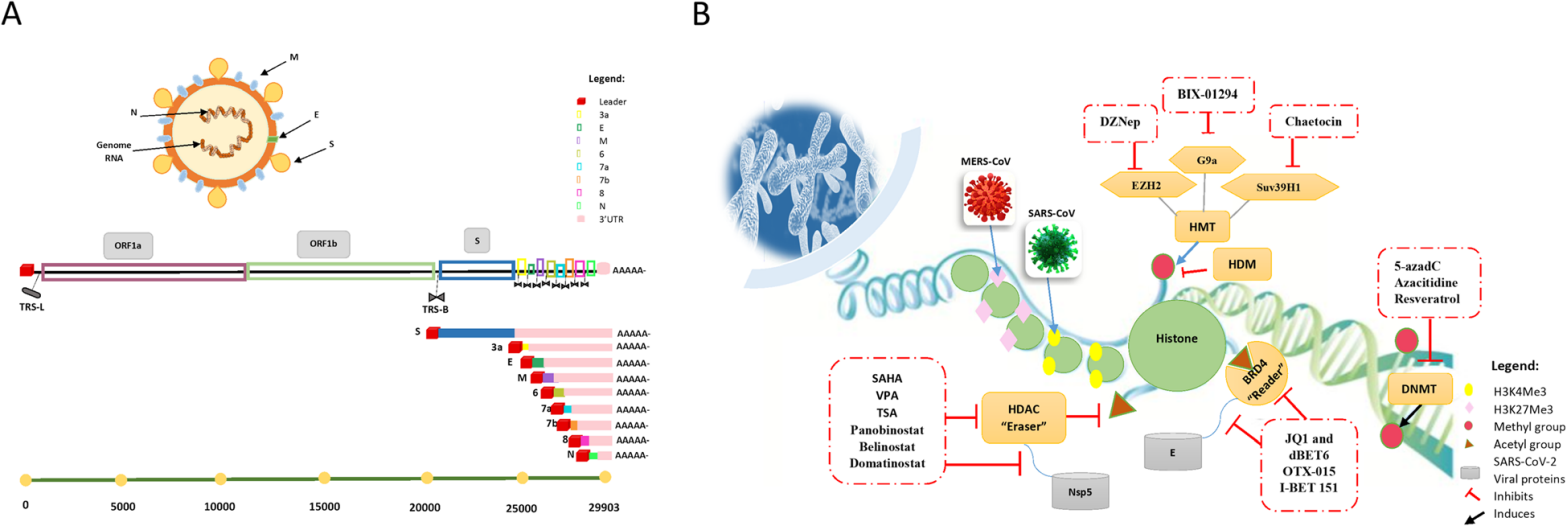
Epigenetics in coronavirus replication and targeted therapies. **a** SARS-CoV-2 genomic map, canonical subgenomic RNAs, and virion structure. Both ORF1a and ORF1b are translated from the genomic RNA. Eight subgenomic RNAs are formed in addition to the genomic RNA. **b** Epigenetic marks and their therapeutic control in HCoV infection. This figure illustrates the epigenetics landscape during coronavirus replication through representing various epigenetic targets (DNMT, HDM, HDAC, and HMT) and their regulation by epigenetic therapy (HDACi, DNMTi, and HMTi). Inhibiting some epigenetic targets leads to a decrease in viral replication load; thus, acting as a vital therapeutic strategy in treating coronavirus infected patients. MERS-CoV in contrast to SARS-CoV increases the repressive epigenetic mark H3k27me3 and decreases the active mark H3k4me3 thus impeding transcription and expression of ISGs. BRD4, a bromodomain and extraterminal (BET) protein, is involved in histone acetylation; besides, it binds to protein E of SARS-CoV-2 accelerating the latter’s entry. Nsp5 binds to HDAC2 and inhibits its entry into the nucleus affecting IFN response

The genome of SARS-CoV-2 is composed of a single-stranded positive RNA of 29 kb; it is considered the largest of all RNA virus genomes (Fig. 1a) [3, 11]. So far, 14 open reading frames (ORF) have been described in the SARS-CoV-2 genome [11, 19]. SARS-CoV-2 genome encodes for viral proteins involved in viral replication named non-structural proteins (Nsp) including the replicase complex coded by ORF1ab, and structural viral proteins involved in viral assembly including the spike (S), envelope (E), membrane (M), and nucleocapsid (NP) protein [3, 11]. The S protein, a class I fusion glycoprotein, forms homotrimers bulging in the viral surface facilitating the viral envelope binding to host cells by attraction with angiotensin-converting enzyme 2 (ACE2). This transmembrane protein is cleaved by the host cell furin-like protease into 2 subunits labeled S1 which binds to the receptor on the host cell surface and S2 is responsible for fusion activity [1, 3]. Hence, disparities in the S protein would directly impact the viral biological characteristics including pathogenicity and antigenicity. Spike protein has been considered as the ultimate target for COVID-19 immunotherapies, and this is based on SARS-CoV and MERS-CoV preceding evidence. Recently, studies have found that SARS-CoV S protein induced polyclonal antibody responses and counteracted SARS-CoV-2 S-mediated entry into host cells, thus favoring the use of this ideal molecular target for vaccination and immunotherapies [20]. Even though SARS-CoV-2 crisis is noteworthy, referring to HCoVs’ data is eminently required since HCoV and SARS-CoV-2 share genomic and biological properties.

#### Epigenetic regulation of HCoV entry

ACE2, a significant player in the renin-angiotensin system (RAS), was recognized as vital factor that attaches to spike protein and eases SARS-CoV-2 binding and host cell entry. Early studies have shown that this receptor acts as a protective mechanism to block the early stages of COVID-19 [11, 21]. ACE2 is significantly expressed in the lower respiratory tract such as type II alveolar cells (AT2) of the lungs, upper esophagus, and stratified epithelial cells, as well as in kidney proximal tubule cells, bladder urothelial cells, absorptive enterocytes, cardiomyocytes, and cholangiocytes. Such cellular outspreading elucidates the consequences of SARS-CoV-2 infection that is not only limited to respiratory disorders but also to kidney, liver, heart, and gastrointestinal tract illnesses [19]. Sirtuins, a family of nicotinamide adenine dinucleotide (NAD)+ dependent deacetylases, play an important role in cellular homeostasis [22]. Silent information regulator T1 (SIRT1), a histone deacetylase (HDAC) class III, is a key regulator of ACE2 levels via binding to its promoter [23]. In fact, resveratrol a well-known activator of SIRT1 increases the ACE2/angiotensin 1-7 (Ang1-7)/Mas receptor (MasR) axis parallel to the downregulation of the Angiotensin II receptor type 1 (AT1R) expression belonging to the prorenin receptor (PRR)/ACE/angiotensin II (Ang II)/AT1R axis [24–28]. Although it was not shown so far that SIRT1 increases SARS-CoV-2 entry into the cell due to increased ACE2 expression, COVID-19 patients with high levels of ACE2 have a better prognosis probably due to decreased hyperinflammation [28–30]. Similarly, SIRT1 could impact viral entry of SARS-CoV and HCoV-NL63 which also use ACE2 for viral entry [31]. Foremost host cellular receptors significantly utilized by other HCoVs for entry and viral replication are aminopeptidase N (APN) by HCoV-229E, dipeptidyl peptidase 4 (DPP4) by MERS-CoV and 9-O-acetylated sialic acid by HCoV-OC43 and HCoV-HKU1 [5, 6, 32, 33]. Any mutations in human APN cell surface receptor will directly inhibit virus-receptor interaction [33]. Promoter hypermethylation downregulates APN gene expression and azacitidine (5-azaC) induces APN protein expression [34]. In melanoma cells, the combination of HDAC inhibitor CHR-3996 and APN inhibitor tosedostat activated synergistically NF-kB [35]. Glucocorticoids directly upregulate the DPP4 gene expression in macrophages due to the presence of two GC-binding motifs in the DPP4 gene promoter [36]. Therefore, the cell surface expression of HCoV entry receptors can be modulated by epigenetic drugs which could be used to discover new therapeutic approaches to curtail HCoV infections.

#### Epigenetic regulation of HCoV replication and transcription

Coronaviruses employ a multisubunit machinery for replication and transcription. The RdRp, also known as Nsp12, catalyzes the synthesis of viral RNA and thus plays a central role in the replication and transcription cycle of SARS-CoV-2, possibly with the assistance of Nsp7 and Nsp8 as cofactors [37]. Nsp7 interacts with the 7SK small nuclear ribonucleoprotein (7SK snRNP) complex comprising La-related protein (LARP7), methyl-phosphate capping enzyme (MEPCE), and hexamethylene bisacetamide inducible protein (HEXIM1). This complex sequesters positive transcription elongation factor (P-TEFb) which is critical for the replication of several viruses including herpesviruses, human immunodeficiency viruses (HIV), human T-lymphotropic virus (HTLV), human adenovirus (HAdV), influenza A virus and Dengue virus (DENV) [38]. The interaction between Nsp7 and P-TEFb will result in the release of the active form of P-TEFb known to bind bromodomain-containing protein 4 (BRD4); similar interaction has been reported previously for HIV Tat and P-TEFb. Interestingly, bromodomain and extra-terminal motif (BET) inhibitors such as JQ1, I-BET, I-BET151, OTX015, UMB-136, MMQO, CPI-203, RVX-208, PFI-1, BI-2536, and BI-6727 induce P-TEFb release and have been reported to be latency reversal agents in HIV infection [39]. Nsp14, a 3′-5′ exonuclease, is critical for coronavirus RNA synthesis [40]; Nsp14 interacts with SIRT5 acting as a weak deacetylase but with desuccinylase and demalonylase activities regulating several metabolic pathways [41]. Recently, SIRT5 inhibitors have been developed [42]. Nsp13 helicase/triphosphatase participate in the release of newly synthesized RNA strand and thus the production of infectious viral particles [43]. Since p300 expression is under HDACs’ control [44], helicases such as Nsp13 might be under the control of p300 and thereby HDAC inhibitors might interfere with coronavirus replication. The Nsp3-Nsp4-Nsp6 complex is also involved in viral replication. Remarkably, Nsp4 interacts with HDAC2, and this could emphasize the role of epigenetic therapies in blocking HDAC2 [45]. Nsp10 is essential for the RNA cap methyl transferase of Nsp16. During RNA capping, Nsp16 methyl transferase activity coud be blocked by methyltransferase inhibitors such as sinefungin, dAPPMA 2′-O MTase inhibitors, aurintricaboxylic acid and inhibitor 7 [46]. Finally, Nsp15 is a uridine-specific RNA endonuclease which has been shown to cleave a highly conserved RNA structure in the 3′ non-translated region of the SARS virus [47]. Most recurrent transcription regulating sequence (TRS) related to MERS-CoV is TRS2. Generally, MERS-CoV transcribes 11 subgenomic mRNAs, including various ORFs translating polypeptide chains and fusion proteins. Unlike MERS-CoV, SARS-CoV includes TRS3 that exists also in SARS-CoV-2. SARS-CoV possesses several accessory proteins (AP), which are translated directly from mRNAs. SARS-CoV-2 grants comparative differences in transcription and subgenomic mRNAs translation compared to other beta-coronavirus. SARS-CoV-2 initiates transcription in 20 TRS sites, transcribing much more forms of subgenomic mRNAs than SARS-CoV and MERS-CoV; this could correlate to the severity and high infectivity of COVID-19 on host patients. SARS-CoV-2 produces around nine forms of fusion proteins, with higher concentrations of Nsp12, Nsp3, Nsp5, and AP12 [48].

#### Epigenetic regulation of HCoV protein maturation

Newly synthesized viral polyproteins are cleaved by viral proteases, namely, the 3C-like protease (3CLpro) and the papain-like protease (PLpro) [49]. ORF1a encodes the two cysteine proteases, 3CLpro and PLpro. While PLpro cuts the first three cleavage sites of its polyprotein, 3CLpro is responsible for cleavage of the residual 11 locations resulting in release of a total of 16 Nsp in both SARS-CoV and MERS-CoV [45]. Upon comparing SARS-CoV and SARS-CoV-2, correspondences have been detected in 5′UTR and 3′UTR regions, protease cleavage positions and amino acid conformation of both structural and non-structural proteins. Yet, the number of spike precursors and specificity of accessory proteins differ in SARS viruses. SARS-CoV processes two spike precursors (S1p and S2p), while SARS-CoV-2 and MERS-CoV produce only one. AP11 is characteristic for SARS-CoV, whereas SARS-CoV-2 translates a specific accessory protein AP12 [48]. Nsp5, the coronavirus 3CLpro, is critical for the maturation of non-structural SARS-CoV-2 proteins. Actively, 3CLpro binds to HDAC2 and tRNA methyl transferase 1 (TRMT1) resulting in their cleavage [45]. Thus, 3CLpro mimics HDAC2 inhibitor and might therefore facilitate viral and/or cellular gene expression in infected cells. TRMT1-catalyzed tRNA modifications are required for redox homeostasis to ensure proper cellular proliferation and oxidative stress survival [50]. Since TRMT1 is cleaved by 3CLpro, SARS-CoV-2 might interfere with tRNA methylation in infected cells. Numerous drugs have been reported to inhibit the activity of 3CLpro including the anti-HIV drug lopinavir [16]. The structural viral proteins (NP, M, E, and S) participate in the formation of new virions across the endoplasmic reticulum. SIRT1 activation following treatment of the infected cells with resveratrol results in decreased viral replication [51]. The viral envelope E binds to BRD2 and BRD4 and could be the target for BET inhibitors [39, 45]. Therefore, blocking SARS-CoV-2 structural proteins’ maturation could result firstly from the usage of SARS-CoV-2 protease inhibitors to annihilate the viral polyprotein cleavage by 3CL pro and/or PLpro, and secondly from the epigenetic treatments to decrease the amount and/or activity of structural viral proteins.

#### Epigenetic-targeted therapies controlling coronavirus replication

Understanding fundamental elements of epigenetic regulation is progressively contributing to concepts of viral epigenetic therapeutics. Epigenetic therapies are among the most active areas of research and this is because of their potential specific targeting mechanisms compared to the conventional therapies. Manipulating gene regulatory networks using epidrugs could be associated with a risk generating from the systemic effect of such therapies. Yet, knowing that HCoVs including SARS-CoV-2 may infect more than one cell type it is advantageous to rely on systemically acting agents. Besides, some proposed epigenetic treatments can be site-directed and consequently minimize unwanted systemic risk. Furthermore, it is imperative to spotlight the SARS-CoV-2 direct RNA sequence data including RNA modifications, the viral transcriptome and epitranscriptome as it could anticipate novel-targeted strategies for the management of SARS-CoV-2 infection [52]. Antiviral therapies targeting the pathogen itself act by inhibiting enzymes responsible for viral genome replication, the viral assembly process, and preventing viral entry by blocking the virus-host receptor binding. Remdesivir, favipiravir, ribavirin, galidesivir, and its salt form (BCX-4430) targeting RdRp; ritonavir and darunavir targeting viral proteases (3CLpro or PLpro) in addition to camostat mesylate which is a serine protease inhibitor directly acting on SARS-CoV-2. Moreover, the FDA approved lopinavir/ritonavir as a target of the coronavirus protease enzyme and arbidol (umifenovir) targeting ACE2 and S protein showed efficacy against SARS-CoV-2 as well as chloroquine which possesses an antiviral and an anti-inflammatory effect [16]. In the primary stages of SARS-CoV epidemic, an empirical treatment of broad spectrum antiviral agents and immunosuppressive doses of steroids were used along with the supportive treatment [53]. Despite the available treatment options, studies documenting the therapies’ efficacy are lacking especially for the SARS-CoV-2 outbreak [11, 54–56]. Nevertheless, extending the choices of treatment by generating epigenetic therapies would provide a real improvement to healthcare community struggling to cope during an outbreak of emerging viral infections (Table 1). Histone deacetylases (HDACs) resemble a main epigenetic target for the treatment of viral infections [64, 65] (Fig. 1b). Hence, HDACs class III such as SIRT1 and resveratrol have been previously described as antiviral effectors [22]. Resveratrol was found to modulate SIRTs’ activity (SIRT 1,2,3,5) especially as a SIRT1 activator, 5' AMP-activated protein kinase (AMPK), protein kinase C, NF-kB, p53, activator protein 1 (AP-1), early growth regulator 1 (EGR-1), sterol regulatory element-binding protein 1 (SREBP-1), and DNA methyltransferase (DNMT)1 thus targeting the regulation of viral infection [22, 57]. Significant downregulation of apoptosis induced by MERS-CoV, prolonged cellular survival post-MERS-CoV infection, potential decrease in viral NP protein expression, and decreased viral replication were detected after resveratrol administration [51]. Besides HDACs class III, the inhibition of the class II HDAC2 is achieved by the approved drug valproic acid (VPA) and the pre-clinical candidate apicidin [45, 59]. VPA displayed minimal control of SARS-CoV-2 growth regardless of drug concentration along with high cytotoxicity levels [45]. In addition to VPA and apicidin, HDAC inhibitors include the broad spectrum trichostatin A (TSA) which reduced the pro-inflammatory mediator’s production, MS-275, and depsipeptide [63, 66, 67]. Other potent HDACi such as vorinostat or suberanilohydroxamic acid (SAHA), belinostat, romidepsin, and panobinostat were already used for the treatment of several cancers such as T cell lymphoma and relapse of multiple myeloma [68–70]. Knowing that viruses depend on host’s epigenetic machinery, some epigenetic drugs used in cancer therapies were shown to give a potential broad spectrum antiviral action against novel emerging viruses. The histone methyltransferases-enhancer of zeste homolog 2/1 (HMT EZH2/1) inhibitors have the ability to attain cellular antiviral state and reduce viral yields instead of inducing activation [68, 71]. A pan-HKMT antagonist, 3-deazaneplanocin-A (DZNep), prove to be therapeutically superior to BIX-01294 which antagonizes HKMT G9a [71]. Chaetocin is a fungal mycotoxin which inhibits HMT Suv39H1, promotes permanent cell cycle arrest, and RNA transcript blockage [72]. Chaetocin is contraindicated with SAHA and JQ-1 as it causes cytotoxicity [73]. On the contrary, a possible synergism was revealed combining BIX-01294 with vorinostat and DZNep with vorinostat [71]. BRD4, a chief gene expression regulator, is involved in recognizing and modifying histone acetylation. Inhibition of BRD4 exhibits an effective antiviral effect against a wide range of RNA and DNA viruses by boosting a potential innate immune response, blocking viral attachment, inducting DNA damage response (DDR), decreasing viral replication, and arresting cell-cycle with no apoptotic signs. Bromodomain and extraterminal protein inhibitors are subdivided into clinical (ABBV-744, CPI-0610, RVX-208) and preclinical (dBET6, JQ-1, MZ1) candidates [45, 74]. By hindering the communication of BRD4 and transmembrane E protein, JQ-1, and dBET6 effectively inhibit the replication of SARS-CoV-2 genome [45, 75]. JQ-1 exhibited a potential viral inhibition at low doses; dBET6 expressed lower cytotoxicity levels than JQ-1. Knowing that the bromodomain and extraterminal protein inhibitors possess varying cytotoxicity further drug assessments must be performed prior patient use [45].

**Table 1:** Epigenetics involved in coronavirus infection and their therapeutic control

Epigenetic drug target		Antagonism “inhibitors”		Potential outcome	Combination therapy(few require clinical validation)
HDAC	Pan-HDAC class I and II	VPA		Affecting inflammatory functions and interferon response [45].	VPA + antivirals (remdesivir, ribavirin, favipiravir, galidesivir) or (rapamycin, selumetinib, trametinib) or prostatin
		TSA		Reduced pro-inflammatory mediator’s production and increased IL-10 production [55].	TSA + antivirals or (rapamycin, selumetinib, trametinib)
		Vorinostat (SAHA)		Diminished genomes initiating gene replication, and induced the expression of cellular proteins responsible for viral inhibition [57].	SAHA + antivirals or (rapamycin, selumetinib, trametinib) or prostatin or BIX-01294 or DZNep
	All HDAC classes including class I, II, and IV	Panobinostat		Affecting EGFR/HER2 signaling, MAPK signaling, PI3K-Akt, and NFκB pathway [58].	Panobinostat + antivirals or (rapamycin, selumetinib, trametinib)
		Belinostat and domatinostat		Enhanced TGF-β expression [58].	Belinostat or domatinostat + antivirals or (rapamycin, selumetinib, trametinib)
HKMT	Pan-HKMT EZH2	DZNep		Attained cellular antiviral state and reduced viral yields [57].	DZNep + vorinostat
	HKMT G9a	BIX-01294		Enhancing antiviral state [57].	BIX-01294 + vorinostat
	HMT Suv39H1	Chaetocin		Permanent cell cycle arrest and RNA transcript blockage [59].	
HAT		Anacardic acid, MG149, C646		Suppressed IL-6 levels [60, 61].	
DNMT		Resveratrol		Down-regulation of apoptosis, decrease in (N) protein expression, and RNA viral replication antagonism [51].	
		Decitabine (5-azadC)		Counteracting hyper-inflammation: lowering pro-inflammatory cytokines (TNF-α, IL-6, IL-1β) and chemokines, inducing IL-10 marker and TGF-β [58].	5-azadC + antivirals or (rapamycin, selumetinib, trametinib)
		Azacitidine		Viral mimicry [62].	Azacitidine + antivirals or (rapamycin, selumetinib, trametinib)
BET proteins (BRD4)		Clinical	ABBV-744, CPI-0610, RVX-208	BRD4 inhibition boosts a potential innate immune response, blocks viral attachment, inducts DNA damage response (DDR), decreases viral replication, and arrests cell-cycle with no apoptotic signs [63].	
		Preclinical	dBET6, JQ-1, MZ1		

### Epigenetic regulation of cellular and immune landscape during coronavirus infection

#### Cellular and immune landscape during coronavirus infection

In the course of viral infections, innate immune cells initiate a transcriptional signal that is cell and stimulus specific. Several main performers of innate immunity, such as signal transducer and activator of transcription 1 (STAT1), myeloid differentiation primary response gene 88 (MyD88), *Toll-like receptor (*TLR)4, TLR7, and TLR3/TIR-domain-containing adapter-inducing interferon-beta (TRIF) diminish infection severity during HCoV infection in vivo. Moreover, interferons (IFN-alpha, IFN-beta, IFN-gamma) which are regulated by histone marks aid in controlling HCoV infections in vivo and in vitro. IFN and tumor necrosis factor (TNF) are the primary response genes of the innate immune system whose promoters show poised promoter features. H3K4me3, H3k27me3, and H3k9me2 are responsible for modulating the activation of the main player IFN. H3K9me2, a repressive histone mark, contributes to DNA methylation and heterochromatin formation and hence stopping histone tail acetylation by recruiting the transcriptional repressor of the heterochromatin protein 1 family. H3K4me3, a histone modification enriched in promoter regions, regulates Toll-like receptors (TLRs). As a result, IFN and innate immune responses are subject to epigenetic regulation mediated by specific epigenetic marks, the operation of histone modification enzymes, DNA methylases, and chromatin remodeling complexes. CoVs have progressed genetic functions that antagonize or delay pathogen recognition as well as IFN sensitive gene (ISG) effector functions. Upon the activation of danger sensors, dendritic cells (DCs), and macrophages a temporal and spatial response is epigenetically initiated. The ability of their epigenome to change within minutes after a stimulus is essential for initiating a speedy antiviral host response and to ensure a persistent/specific defense response. Hence, epigenetic mechanisms are responsible guaranteeing a functional and highly regulated host response beyond the initial activation wave [18, 76]. Several epigenetic factors were shown to be effective in the activation of immune responses: recruitment of transcription machinery, prevention of undesired expression of compelling mediators, and repression or stimulation of secondary gene programs [18]. Upon utilizing chromatin immuno-precipitation (ChIP) PCR approaches, it is possible to determine differential occupancy of histone marks at the promoters of ISG genes. During SARS-CoV infection, the promoter regions of ISG genes had more histones with active marks of H3K4me than the repressive H3K27me3 mark, therefore favoring open chromatin and promoting active transcription and ISG expression. MERS-CoV infection of Calu3, a continuous human airway epithelial cell line, resulted in increased levels of H3K27me3 and depletion of H3K4me3 occupancy at the promoter regions of subsets of specific ISGs. These viruses favored a closed chromatin conformation that inhibits ISG expression, which rather was regulated by epigenetic control mechanisms [9, 18, 76]. Thus, the setting of histone methylation marks could be different in two close CoV members, namely SARS-CoV and MERS-CoV.

Acute respiratory distress syndrome (ARDS) is a common immunopathological event for SARS-CoV, MERS-CoV, and SARS-CoV-2. A massive cytokine storm is considered as one of ARDS’ principle mechanisms which is the chief death cause of COVID-19 in addition to MERS-CoV and SARS-CoV severe diseases [14]. During the cytokine storm, pro-inflammatory cytokines (IL-1β, IL-6, IL-12, IL-18, IL-33, TNF-α) and chemokines (CCL2, CCL3, CCL5, CXCL8, CXCL9, CXCL10) are upregulated by effector cells in SARS-CoV infection linked with respiratory distress [14, 77–79]. Unlike the proinflammatory cytokine IL-6, the anti-inflammatory Th2 cytokine TGF-β is not overproduced in COVID-19 patients [77]. Augmented levels of IL-2, IL-6, IL-7, IL-10, granulocyte-colony stimulating factor (G-CSF), TNF-α, IFN-γ-inducible protein 10 (IP10), macrophage inflammatory protein 1-α (MIP1A), and monocyte chemoattractant protein (MCP1) were associated with COVID-19 severity; a cytokine outline favoring secondary hemophagocytic lymphohistiocytosis (sHLH) [80, 81]. Poor prognosis of SARS-CoV-2 infected patients can be forecasted by SARS-CoV-2 viral load which is concomitant with cytokine storm caused by the extremely high IL-6 levels [82]. Older, chronically ill and immunocompromised patients are expected to be more susceptible to hyperinflammation. Demethylation of IFN-regulated genes, NF-kB, and key cytokine genes favors the expression of proinflammatory cytokines and chemokines thus increasing cytokine storm incidence [83]. As a result, minimizing IL-6 plasma concentrations and controlling ACE2 gene epigenetically might be a target for prevention and therapy in COVID-19 [82, 83]. In contrast to SARS-CoV-2, delayed serum levels of IFN, IL-1β, IL-6, IL-8, CCL-2, CCL-3, CCL-5, and IL-2 were detected in MERS-CoV-infected patients. Recent data revealed that pro-inflammatory cytokines (IL-6) and chemokines (IL-8, CXCL-10, and CCL5) responses were elevated in severe MERS-CoV cases [14, 78]. Low levels of the anti-inflammatory cytokine IL-10 was seen in confirmed SARS cases with severe disease. It is noteworthy that SARS-CoV infects monocyte-macrophages, DCs, and T cells which produce IL-10 [78]. In addition to the role of immune cells, the immune landscape in the lung of COVID-19 patients can also be shaped by SARS-CoV-2 infection of the airway epithelial cells. Thus, prominent epigenetic regulators including HDAC2 and BRDs (BRD2, BRD4) interact with viral proteins Nsp5 and E, respectively. Nsp5 antagonizes HDAC2 transport into the nucleus; therefore, affecting HDAC2-induced inflammatory functions and IFN response [18, 45]. Altogether, the hyperinflammatory cytokine and chemokine storm observed in ARDS patients result from epigenetic modifications present in both HCoV infected epithelial cells and immune cells present in their vicinity.

#### Epigenetic treatments controlling immune hyperactivation

**Fig. 2:**
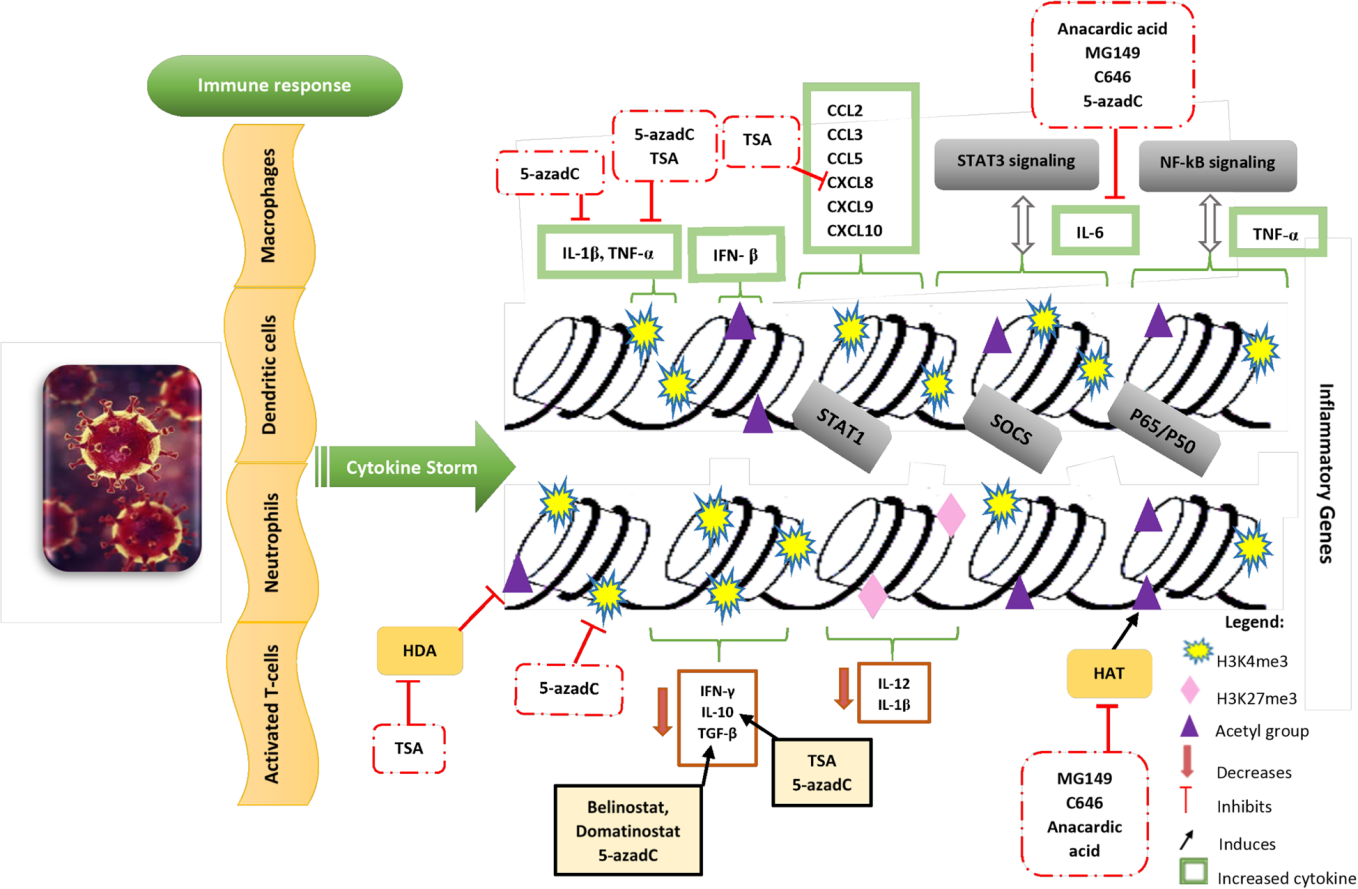
Epigenetics in COVID-19 immune syndrome and targeted therapies. Cytokine storm occurred in the majority of severe COVID-19 cases; hypercytokinemia post-coronavirus infections can be harmful and sometimes deadly. Activated immune cells (T cells, DCs, macrophages, and neutrophils) act as the main immunity system performers. Main pro-inflammatory cytokines (IL-1β, IL-6, IL-12, TNF-α) and chemokines (CCL2, CCL3, CCL5, CXCL8, CXCL9, CXCL10) are upregulated by effector cells due to hyper-methylation and acetylation modifications taking place on histone marks. The increase in repressive histone mark led to a decrease in IL-12 and IL-1β. Epigenetic interventions such as HDACi, HATi, and DNMTi targeted both pro-inflammatory and anti-inflammatory cytokines (IL-10 and TGF-β). The ultimate goal of such interventions is to upregulate anti-inflammatory cytokines and to deplete pro-inflammatory cytokines’ levels through epigenetic modulation

Epigenetic interventions must be urgently directed against cytokine storm to avoid or reduce ARDS and rising mortality (Fig. 2). Targeted anti-cytokine methodologies have proven efficacious in handling cytokine storm syndrome [77]. Dexamethasone, an effectual corticosteroid, was shown recently to decrease the cytokine storm syndrome with reduced mortality rates in COVID19 patients [84, 85]. IL-1 inhibitor anakinra and the IL-6 receptor inhibitor tocilizumab proved a remarkable survival benefit in patients experiencing hyperinflammation post-SARS-CoV-2 infection [80, 81]. IFN-αβ and IFN-γ inhibitors mitigate HCoV-induced inflammation; the timing of IFN antagonism therapy must be taken into consideration as it defines the disease outcome. An early IFN response was protective in SARS-CoV-infected mice unlike a delayed IFN signaling. During delayed severe stages of SARS-CoV, IFN-αβ receptor antagonists prevent inflammatory responses; IFNγ neutralization limited IFN-α production [78, 79]. Supplementary, anti-TNFα antibody administration significantly dampened IFNγ content [79]. DNA methylation is directed via DNA methylase DNMT3a/b by binding of H3K9 histone methyltransferase G9a to the TNFα promoter which will then render TNFα promoter in a transcriptionally repressive state, resulting in reduced TNFα protein levels. Histone acetylation (H3K9ac, H3K36ac, and H4K5ac) triggers the suppression of IL-8 and TNFα levels which were majorly produced in response to CoV. By means of broad spectrum HDACi, TSA, the production of these proinflammatory mediators was curbed [64, 76].

Resveratrol counteracts hyperinflammation by interfering with NF-kB pathway [51]. It potentiates SIRT1 which directly interacts with p65 via deacetylation on lysine 310 resulting in the inhibition of NF-κB activation. Additionally, inhibiting NF-κB activity via SIRT1-AMPK signaling pathway in the cytoplasm promotes an enhanced anti-inflammatory effect. In fact, NF-kB activation is required for the production of numerous proinflammatory cytokines including TNF-α, IL-1β, IL-6, and proinflammatory chemokines. Besides, resveratrol reduces TNF-α-induced phospho-p38 MAPK expression [86–88].

Baricitinib, a JAK inhibitor as well as an AP2-associated protein kinase 1 (AAK1) inhibitor, was approved for inhibiting IFN-α production in hyperinflammatory cases caused by SARS-CoV-2 [81, 89]. Epigenetic drugs targeting proinflammatory and anti-inflammatory cytokines/chemokines act as a beneficial forecaster of long-term outcome for CoVs infections. The aim is to dampen the major proinflammatory cytokines (IL-6, TNF-α, IL-1β) and chemokines (IL-8, MCP-1, CCL5) response while enhancing the role of anti-inflammatory cytokines (IL-10 and TGF-β). Altering DNA methylation and histone acetylation states in the IL-6 promoter will impact IL-6 expression [90–92]. Histone acetyltransferase inhibitors (HATi) such as anacardic acid, MG149, and C646 suppress IL-6 levels [60, 61]. Decitabine or 5-aza-2-deoxycytidine (5-azadC), a nucleoside-based DNMT inhibitor, is widely used to inhibit DNA methylation in macrophages; thus, suppressing inflammation [58, 70, 93]. A recent study suggested that the decrease of suppressor of cytokine signaling 1 (SOCS1) via promoter hypermethylation is strongly associated with overproduction of proinflammatory cytokines (TNF-α and IL-6) illuminating the use of DNMTi [90]. Besides, 5-azadC induced IL-10 marker and TGF-β [58, 70, 93]. Belinostat and domatinostat enhanced the expression of TGF-β [58]. Moreover, treatment with TSA resulted in increased production of IL-10 [63, 67, 69]. Hence, elevation of IL-10 and TGF-β levels might curtail the hyperinflammatory storm observed in COVID-19 patients associated with poor prognosis.

### Combination therapy to fight coronavirus infections including antiviral and epigenetic drugs

Suitable combination therapies reduce the likelihood of drug resistance, suppress viral replication, lower toxicity levels, and provide synergistic effects (Table 1). Achieving high potency intensities, combinatorial treatments were employed especially in RNA viruses which mutate at higher rate than DNA viruses. However, care must be taken in order to avoid unwanted contraindications. Although there is no clinically approved antiviral drug or vaccine available to be used against COVID-19, few therapeutic combinations have been evaluated to cope with this viral outbreak. Some patients were clinically recovered after the administration of remdesivir combined with chloroquine (CQ) or IFN-β due to the significant blockade of the SARS-CoV-2 replication [89]. In Saudi Arabia, a clinical trial discovered that a combination of lopinavir/ritonavir and IFN-β1b was shown to be effective among MERS-CoV-infected patients [12]. It is also hypothesized that the combination of lopinavir/ritonavir and arbidol will deliver enhanced efficacy against SARS-CoV-2 for a synergistic effect is predictable [16]. A higher potency of antiviral therapy was achieved after the fusion of ribavirin and IFN with immunomodulating agents such as intravenous N-acetylcysteine [94]. Few synergistic combinations resulting from a network-based analysis include sirolimus and dactinomycin, an approved RNA synthesis inhibitor, target HCoV-associated host protein subnetwork by “complementary exposure” pattern, resulting in potential combination regimens for treatment of HCoVs. To successfully inhibit MERS-CoV replication, kinase inhibitors were combined with other host-targeting molecules such as peroxisome proliferator-activated receptor alpha (PPAR-α) agonists [11]. In a retrospective analysis, ribavirin was tested in combination with corticosteroids, immunoglobulins, and/or antibiotics for SARS-CoV; no efficacy and high fatality rates were shown. Since ribavirin treatment did not improve patient outcome health, Canada stopped permitting the use of ribavirin. Additional studies tested the activity of ribavirin jointly with lopinavir against SARS-CoV. Compared to the control groups, confirmed SARS-CoV cases undergo a milder disease progression with no consequences. In another retrospective analysis, patients were treated with oral ribavirin and SC pegylated IFN-α2a for 2 weeks. At day 14 after confirmed diagnosis of MERS, survival was increased in the tested group (70%) compared to the control (29%). In an additional case study, an elderly patient who was infected with MERS in Jeddah was treated with oral lopinavir/ritonavir, pegylated IFN, and ribavirin. Viral RNA was detected in feces, serum, and respiratory secretions’ samples after 2 days for initiating the therapy and up to 2 weeks. An ongoing randomized clinical trial in Saudi Arabia is evaluating treatment of MERS patients with IFN-β1b conjointly with lopinavir/ritonavir due to the latter’s high efficacy [4]. Among clinical treatments studied for treating SARS-CoV, combinations of steroid with either alfacon-1, a recombinant consensus IFN-α, or protease inhibitors and ribavirin were found to improve patients’ health [56]. Patients with COVID-19 are being recruited in randomized trials to evaluate the efficacy of favipiravir plus IFN-α and favipiravir plus baloxavir marboxil (an approved influenza inhibitor targeting the cap-dependent endonuclease). The synergism between a pegylated-IFN and a nucleoside compound against COVID-19 is still ambiguous [16]. In spite of drug repurposing and the use of targeted antiviral therapies (CQ, remdesivir, rapamycin, ribavirin), epigenetic drugs such as BRD4 inhibitors, DNMT1 inhibitors, and HDAC inhibitors have been demonstrated to potentially inhibit SARS-CoV-2 [4, 14, 45, 95]. Novel broad spectrum replication inhibitors such as remdesivir or GS-5734 (Gilead Sciences, in phase I clinical trial), and immunomodulators along with direct-acting antiviral agents that are in development could make an efficient amalgamation for treating HCoVs [4]. Knowing that the core treatment for eradicating HCoVs is controlling replication and immune response, offering drug therapies that target both pathways could be the best approach. Some epigenetic therapies have a dual action; resveratrol decreased the expression of NP protein in addition to SIRT-1 activation which counteracts viral replication and hyperinflammation [51]. The anti-viral and anti-inflammatory effects of CQ may play a crucial role in prevention and treatment of COVID-19 as it operated at entry and post-entry phases of SARS-CoV-2 infection [16, 96–98]. Generally, CQ blocks viral infection by elevating endosomal pH necessary for entry, replication, and maturation. For MERS-CoV and SARS-CoV, CQ interfered with cellular proteases and glycosylation of ACE2, respectively [16, 98]. Along with CQ’s antiviral effect, CQ decreases the production of pro-inflammatory indicators and cytokines reducing cytokine storm destruction [96, 97]. Merging antiviral drugs with epigenetic therapies targeting hyperinflammation is considered as an alternative. Antivirals (remdesivir, ribavirin, favipiravir, and galidesivir) could be combined with DNMTi (decitabine, azacitidine) or HDACi (vorinostat, belinostat, panobinostat, TSA). However, further preclinical experiments and clinical trials are required to validate the clinical benefits of these combined candidates. Epidemiological studies showed that the majority of severe SARS-CoV-2 cases were elderly patients with comorbid conditions whereas children cases have been rarely reported [3, 54] suggesting the use of anti-aging drugs targeting epigenetics (resveratrol), other anti-aging drugs (CQ, rapamycin, and doxycycline) and senolytics (azithromycin and quercetin) which could decrease substantial morbidity and mortality [99, 100]. Several anti-aging therapeutics exists as FDA-approved drugs with acceptable safety profile proposing their use in COVID-19 prevention [99].

## Conclusion and perspectives

Providing more research to generate infection control therapeutic interventions is a major challenge. The three coronavirus global outbreaks emphasized the exigent need for curtailing CoVs infections despite the presence of many therapeutic options including epigenetic therapy, antivirals, and repurposing drugs. Cationic amphiphilic drugs, analogs of previously developed drugs, antibody therapy, structure-based drug design, and combination therapies are considered as researchers’ key for further novel antiviral drug development [4, 89]. Safe and effective coronavirus vaccine is the ultimate weapon for reducing morbidity and mortality rates. Coronavirus S protein is considered a strategic board for vaccines [89, 101]. Although several vaccination strategies are being tested against CoVs, these studies are still in progress [14, 89]. Live attenuated and deactivated virus, viral vectors, recombinant DNA, subunit, and protein vaccines are considered as the main approaches [14]. Additional clinical and laboratory evidences must be investigated to validate the use of vaccines proposed by several academic institutions [11, 14, 17, 89, 101]. A recent study specified the clinical benefits of the microneedle array (MNA) delivered recombinant protein subunit vaccines as a favorable immunization strategy against SARS-CoV, MERS-CoV, and SARS-CoV-2 [101]. In closing, research efforts must be deepened through all outbreak stages to end coronavirus pandemic and epigenetic-targeted therapy could be a major asset in restricting severe HCoV infections.

## Data Availability

Not applicable.

## Abbreviations

3CLpro: 3C-like protease
5-azadC: 5-aza-2-deoxycytidine
7SK snRNP: 7SK small nuclear ribonucleoprotein
AAK1: AP2-associated protein kinase 1
ACE: Angiotensin-converting enzyme
ACE2: Angiotensin-converting enzyme 2
ALI: Acute lung injury
AMPK: 5' AMP-activated protein kinase
Ang II: Angiotensin II
Ang1-7: Angiotensin 1-7
AP: Accessory proteins
AP-1: Activator protein 1
APN: Aminopeptidase N
ARDS: Acute respiratory distress syndrome
AT1R: Angiotensin II receptor type 1
AT2: Type II alveolar cells
BET: Bromodomain and extra-terminal motif
BRD: Bromodomain-containing protein
CCL/CXCL: Chemokine
ChIP: Chromatin immuno-precipitation
COVID-19: Coronavirus infectious disease 2019
CoVs: Coronaviruses
CQ: Chloroquine
DCs: Dendritic cells
DDR: DNA damage response
DENV: Dengue virus
DNMT: DNA methyltransferase
DPP4: Dipeptidyl peptidase 4
DZNep: 3 Deazaneplanocin-A
E: Envelope
EGR-1: Early growth regulator 1
EZH2/1: Enhancer of zeste homolog 2/1
FDA: Food and Drug Administration
G-CSF: Granulocyte-colony stimulating factor
GS: Gilead Sciences
HAdV: Human adenovirus
HATi: Histone acetyltransferase inhibitors
HCoVs: Human coronaviruses
HDAC: Histone deacetylase
HIV: Human immunodeficiency viruses
HMT: Histone methyltransferases
HPV: Human papilloma virus
HTLV: Human T-lymphotropic virus
HXIM1: Hexamethylene bisacetamide inducible protein
IFN: Interferons
IL: Interleukine
IP10: IFN-γ-inducible protein 10
ISG: IFN sensitive gene
LARP7: La-related protein
M: Membrane
MasR: Mas receptor
MCP1: Monocyte chemoattractant protein
MEPCE: Methyl-phosphate capping enzyme
MERS-CoV: Middle East respiratory syndrome coronavirus
MIP1A: Macrophage inflammatory protein 1-α
MNA: Microneedle array
MyD88: Myeloid differentiation primary response gene 88
NAD: Nicotinamide adenine dinucleotide
NP: Nucleocapsid
Nsp: Non-structural proteins
ORF: Open reading frames
PHEIC: Public Health Emergency of International Concern
PLpro: Papain-like protease
PPAR-α: Peroxisome proliferator-activated receptor alpha
PRR: Prorenin receptor
P-TEFb: Positive transcription elongation factor beta
RAS: Renin-angiotensin system
RdRp: RNA dependent RNA polymerase
S: Spike
SAHA: Suberanilohydroxamic acid
SARS-CoV: Severe acute respiratory syndrome coronavirus
sHLH: Secondary haemophagocytic lymphohistiocytosis
SIRT1: Silent information regulator T1
SOCS1: Suppressor of cytokine signaling 1
SREBP-1: Sterol regulatory element-binding protein 1
STAT1: Signal transducer and activator of transcription 1
TLRs: Toll-like receptors
TNF: Tumor necrosis factor
TNFR: Tumor necrosis factor receptor
*TRIF*: TIR-domain-containing adapter-inducing interferon-β
TRMT1: tRNA methyl transferase 1
TRS: Transcription regulating sequence
TRS-B: Transcription-regulatory sequences in the body
TRS-L: Transcription-regulatory sequences in the leader
TSA: Trichostatin A
VPA: Valproic acid
WHO: World Health Organization
